# Supplementary material for: Analysis of the heterogenous structural states of the hexameric ATPase PilU of the type IV pili from Vibrio cholerae
Source: Protein Sci. 2026 May 5;35(6):e70609. doi: 10.1002/pro.70609 (PMC13142100; doi:10.1002/pro.70609)
Supplement: Supplementary file 1 — Movie S1. Morph trajectory of conformational forms 1, 4, 3, 2, 6. Morphing trajectory generated from PDB models illustrating the structural transitions between forms 1, 4, 3, 2, and 6, highlighting the conformational rearrangements and relative domain movements across these states. Movie S2. 3D variability analysis of particle heterogeneity. 3D variability analysis showing the principal modes of conformational heterogeneity (components 0, 1, and 2) within the dataset. Movie S3. Morph trajectory of forms 1, 4, 3, 2, 6, and 5. Morphing trajectory generated from PDB models illustrating the structural transitions between forms 1, 4, 3, 2, 6, and 5. [file PRO-35-e70609-s001.pptx]

## Slide 1
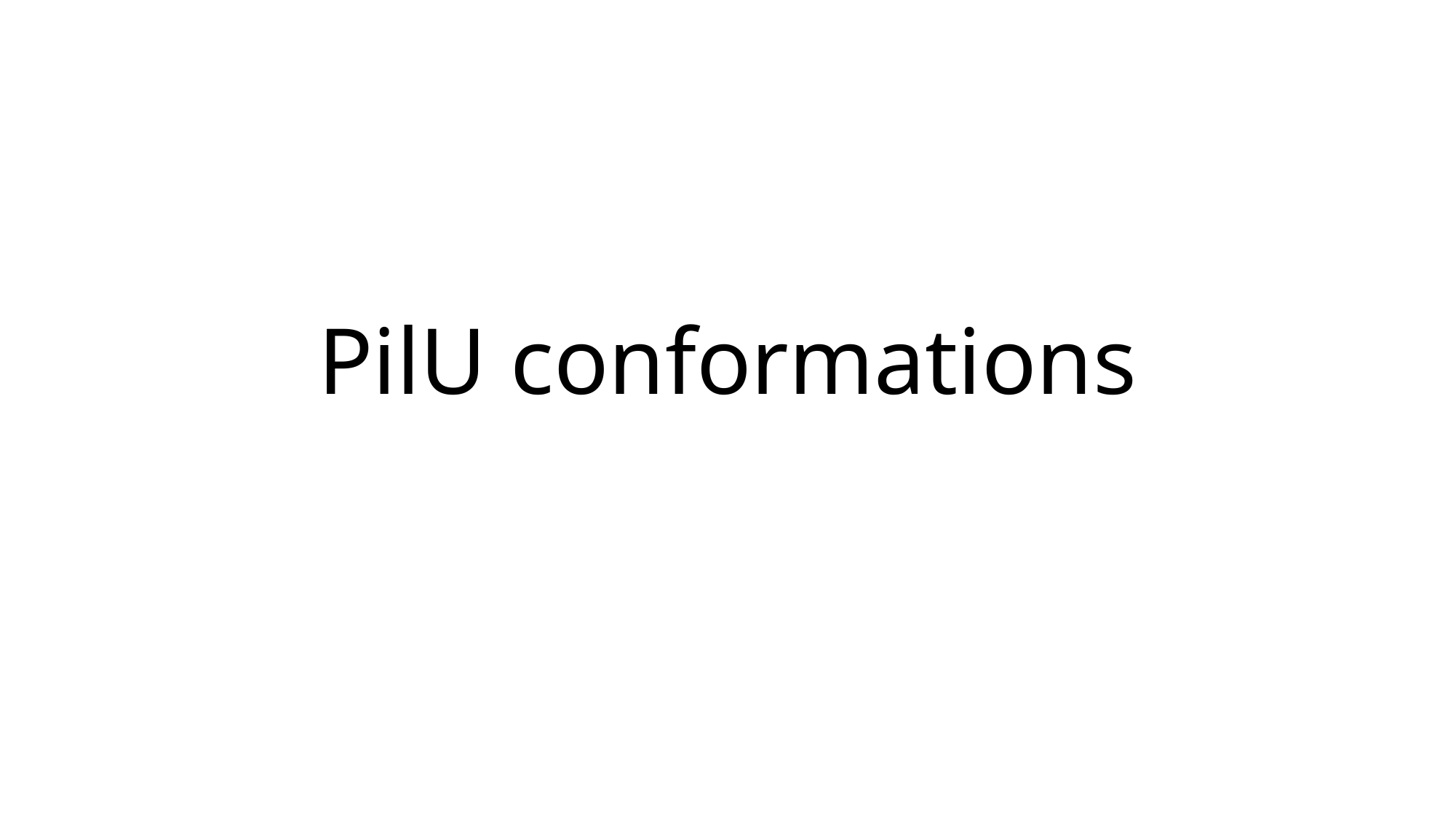

# PilU conformations

## Slide 2
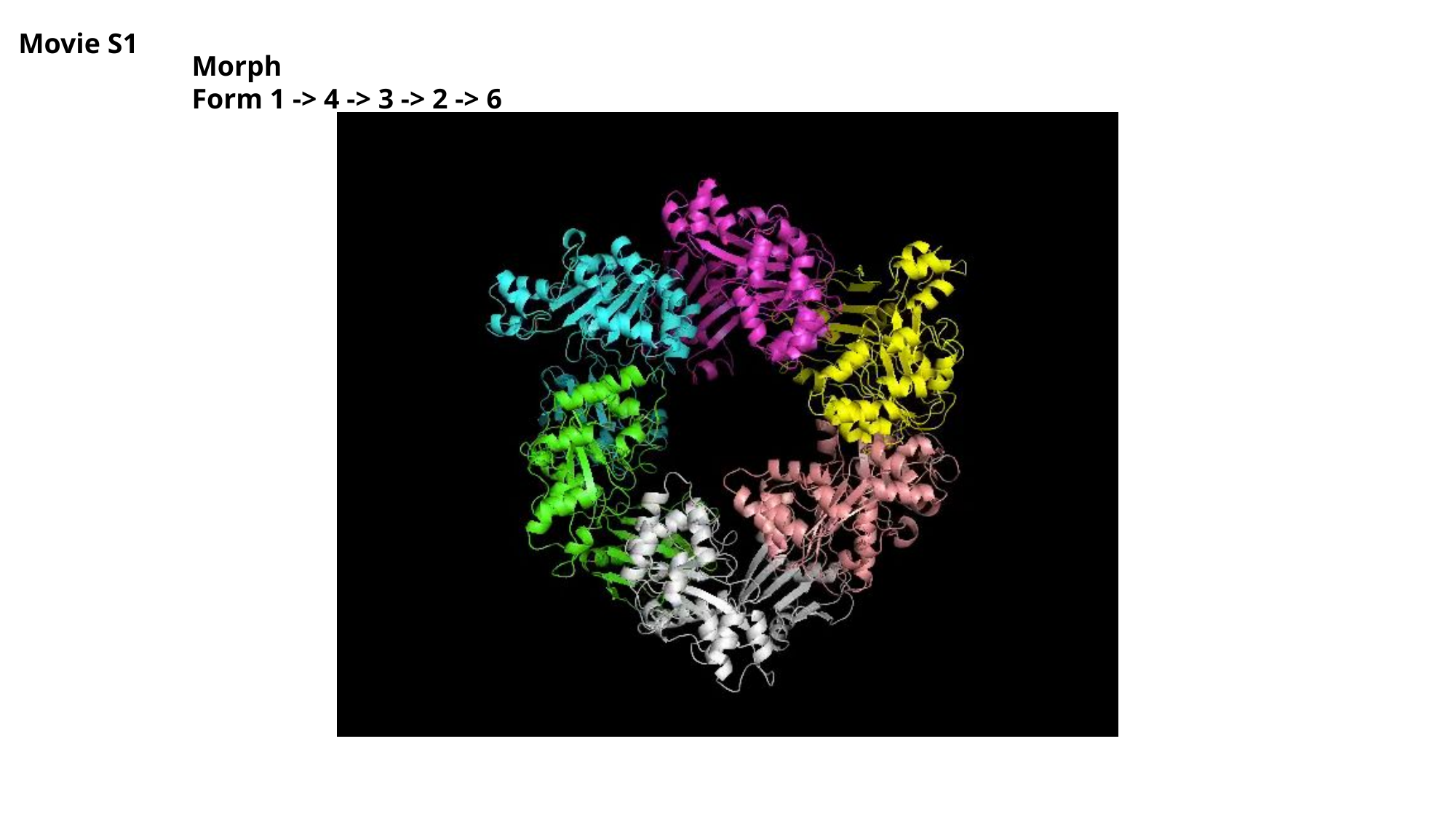

Movie S1
Morph
Form 1 -> 4 -> 3 -> 2 -> 6

## Slide 3
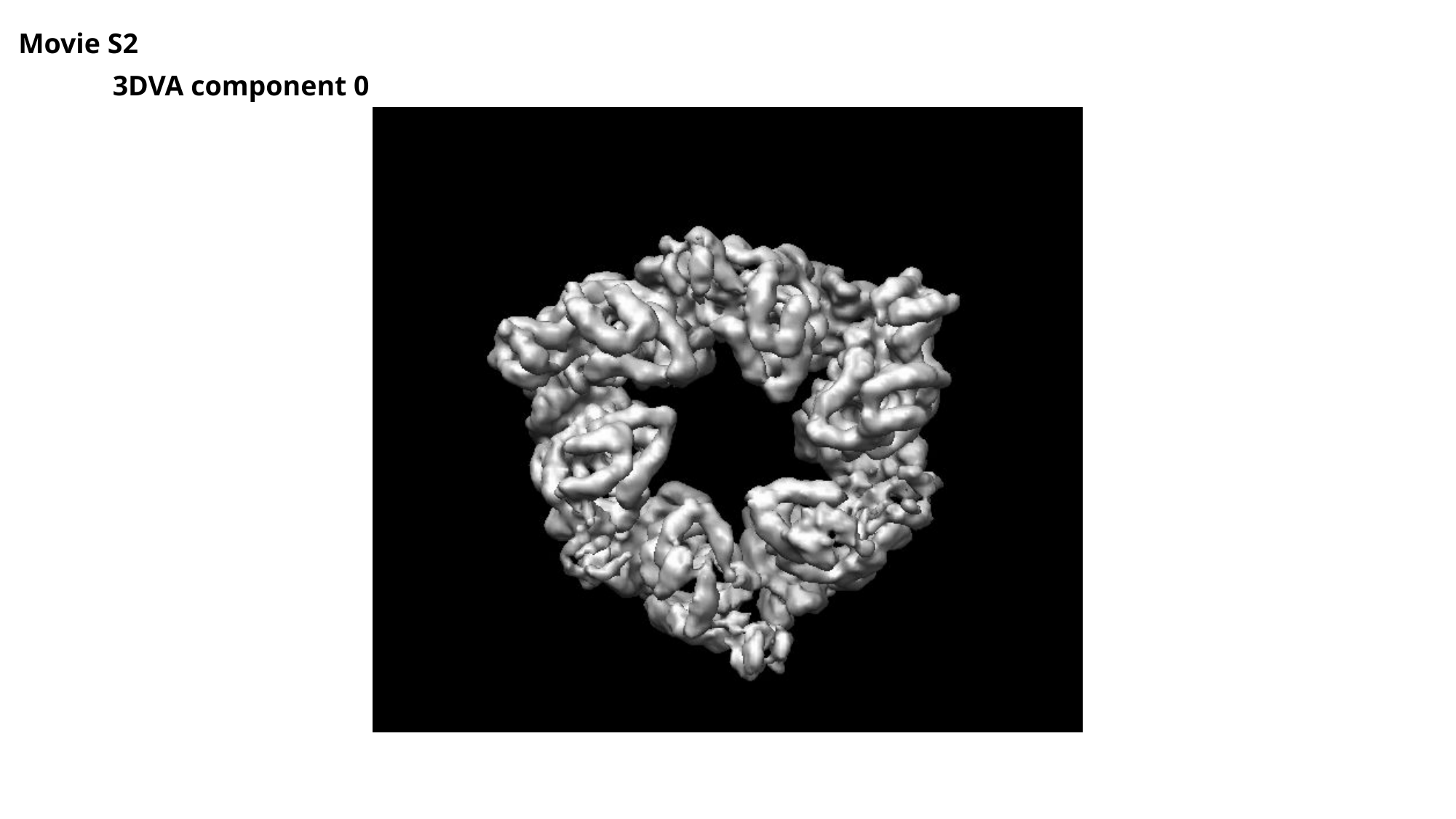

Movie S2
3DVA component 0

## Slide 4
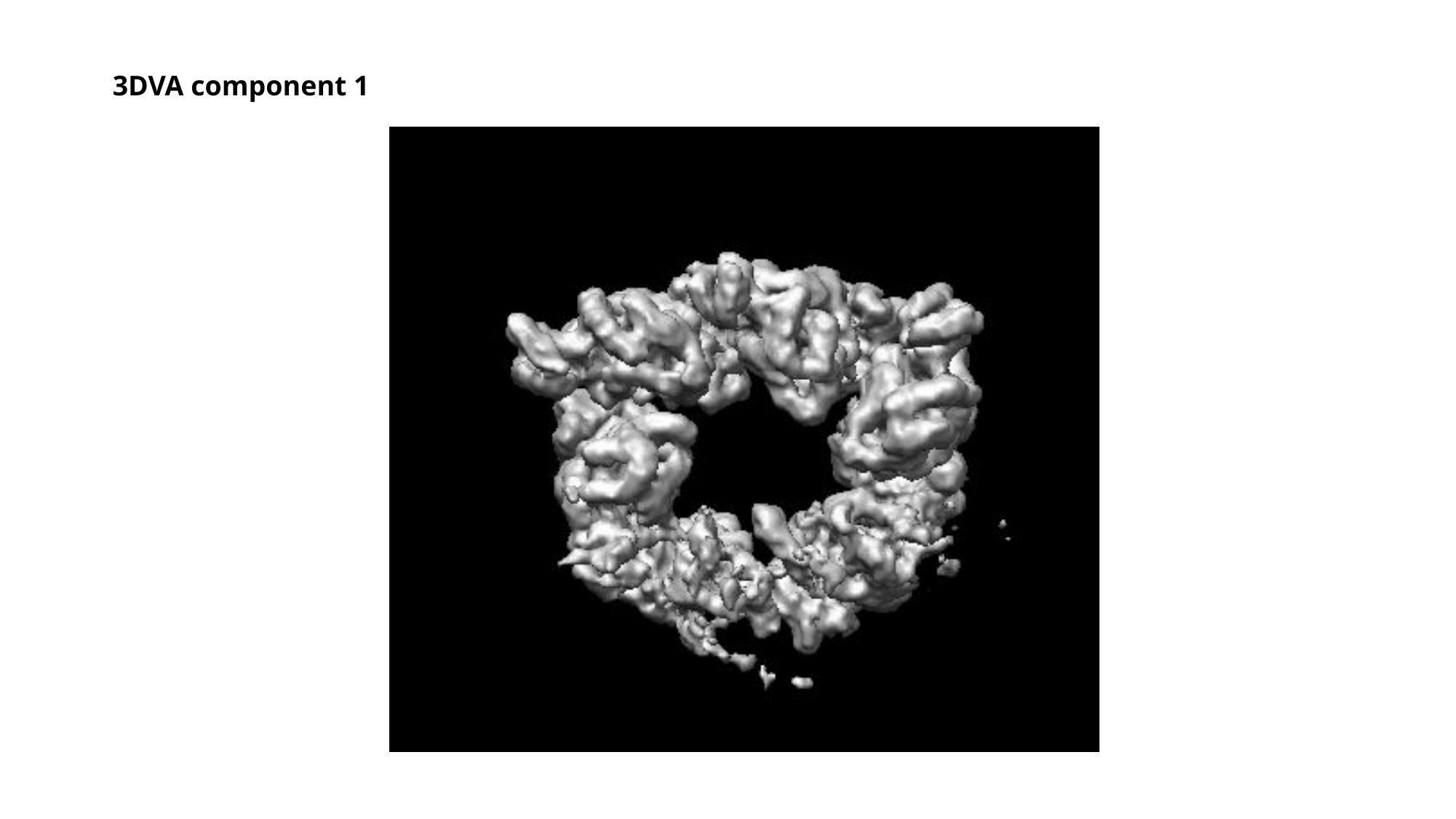

3DVA component 1

## Slide 5
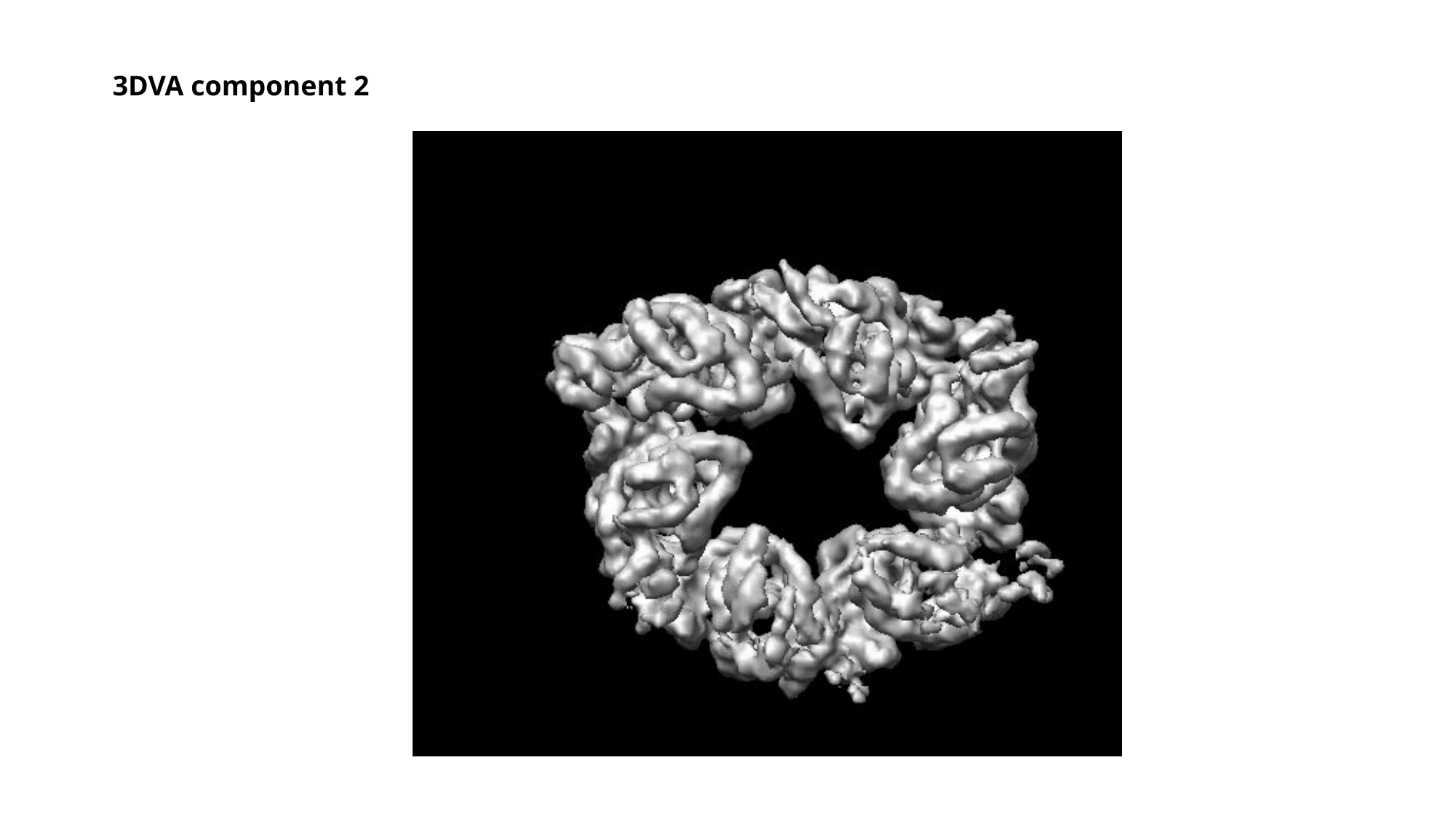

3DVA component 2

## Slide 6
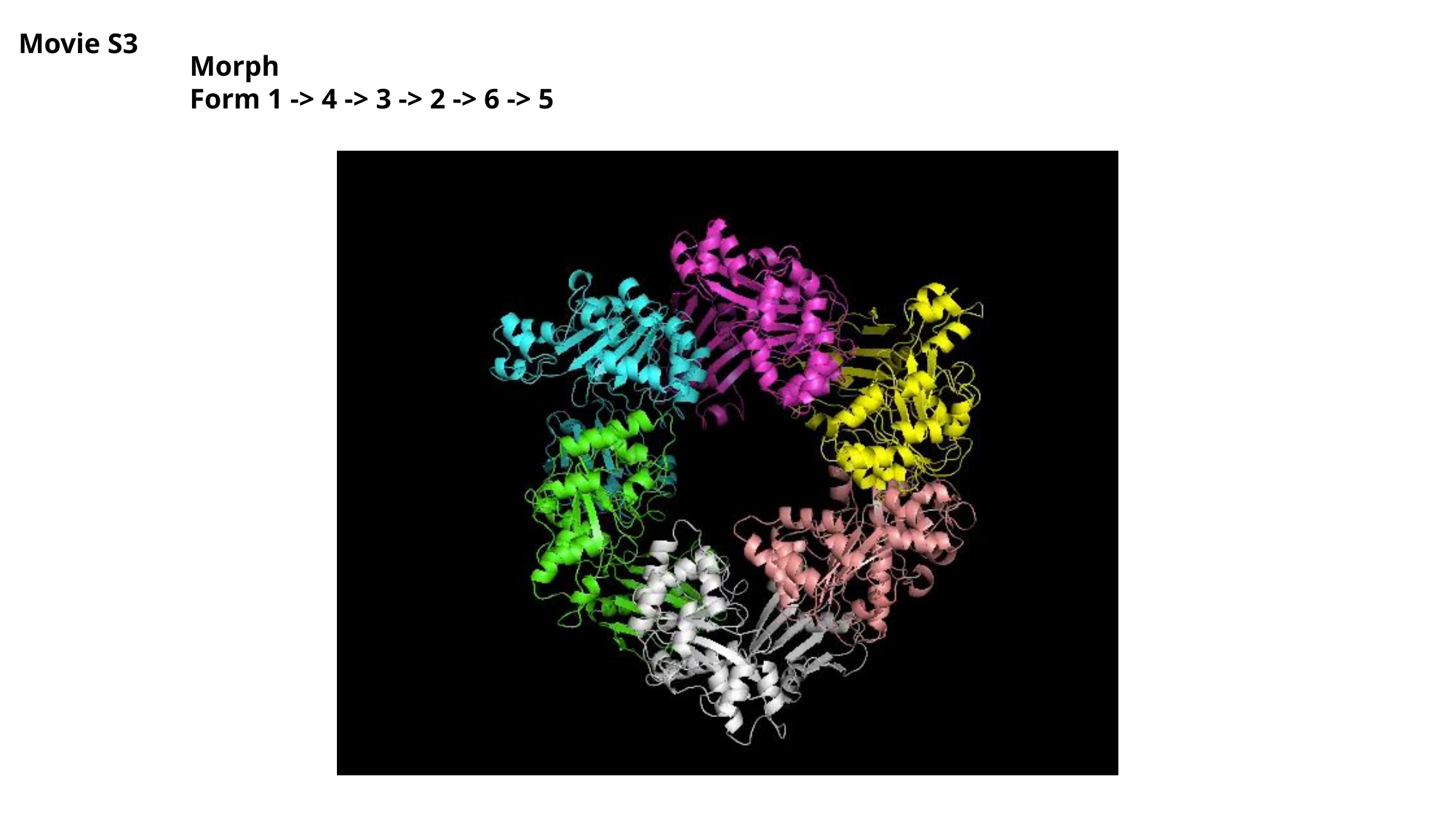

Movie S3
Morph
Form 1 -> 4 -> 3 -> 2 -> 6 -> 5
