# Supplementary material for: Analysis of the heterogenous structural states of the hexameric ATPase PilU of the type IV pili from Vibrio cholerae
Source: Protein Sci. 2026 May 5;35(6):e70609. doi: 10.1002/pro.70609 (PMC13142100; doi:10.1002/pro.70609)
Supplement: Supplementary file 2 — Figure S1. Sequence alignment of PilU, PilT, and PilB. Figure S2. Cryo‐EM particle flow. Three datasets (dataset 1: apo, 3559 movies; dataset 2: in the presence of ATP, 3788 movies; dataset 3: in the presence of ADP, 3836 movies) were collected and processed independently to select clean particles. From dataset 1, 3D classification followed by homogeneous refinement yielded PilU form 1 (C3 symmetry, 3.44 Å, cyan box). Qualitative inspection of 2D class averages showed no obvious nucleotide‐dependent differences in overall particle shape, apparent symmetry, or distribution of major views; therefore, particles from all three datasets were pooled for further analysis. Multiple rounds of 3D classification and homogeneous refinement of the combined particle set resolved PilU form 2 (C2 symmetry, 3.68 Å, red box), form 3 (C2 symmetry, 3.49 Å, blue box), and form 4 (C2 symmetry, 3.76 Å, green box). Similar conformations corresponding to PilU forms 1–4 were observed across multiple datasets and 3D classification jobs (cyan dashed box: classes similar to form 1; red dashed box: classes similar to form 2; blue dashed box: classes similar to form 3; green dashed box: classes similar to form 4), further indicate that the addition of ATP and ADP did not substantially change the overall PilU conformations, even if the relative population of particles in each class may differ. For clarity, only the highest‐quality reconstruction of each form is used for atomic model building and subsequent analysis. 3D reconstructions with nominal resolutions worse than ~4 Å, which may represent minor conformations or other poorly resolved classes, lacked interpretable structural features and were therefore not assigned as discrete PilU forms and were not further analyzed. Differences in particle numbers across similar reconstructions from individual classification jobs likely reflect the stochastic nature of 3D classification in the presence of continuous heterogeneity and its sensitivity to para [file PRO-35-e70609-s002.docx]

**Supplementary information**

**Analysis of the heterogenous structural states of the hexameric ATPase PilU of the Type IV pili from *Vibrio cholerae***

Yirui Guo^1,2^, Shantanu Shukla^3,4^, George Minasov^3,4^, Nicole L. Inniss^3,4^, Thomas Klose^4,5^, Valerie L. Tokars^6,7^, Alfonso Mondragón^8^, Zbyszek Otwinowski^1,9^, Dominika Borek^1,4,9,*^, Karla J. F. Satchell^3,4,*^

^1^ Department of Biophysics, The University of Texas Southwestern Medical Center, Dallas, TX 75390, USA

^2^ Ligo Analytics, Dallas, TX 75206, USA

^3^ Department of Microbiology-Immunology, Northwestern University Feinberg School of Medicine, Chicago, IL 60611, USA

^4^ Center for Structural Biology of Infectious Diseases, Northwestern University Feinberg School of Medicine, Chicago, IL 60611, USA

^5^ Department of Biological Sciences, Purdue University, West Lafayette, IN 47907, USA

^6^ Department of Pharmacology, Northwestern University Feinberg School of Medicine, Chicago, IL 60611, USA

^7^ Present address: Department of Structural Biology, St. Jude Children’s Research Hospital, Memphis, TN 38105, USA

^8^ Department of Molecular Biosciences, Northwestern University, Evanston, IL 60208, USA

^9^ Department of Biochemistry, The University of Texas Southwestern Medical Center, Dallas, TX 75390, USA

*Corresponding authors

Dominika Borek: dominika.borek@utsouthwestern.edu

Karla Satchell: k-satchell@northwestern.edu

**
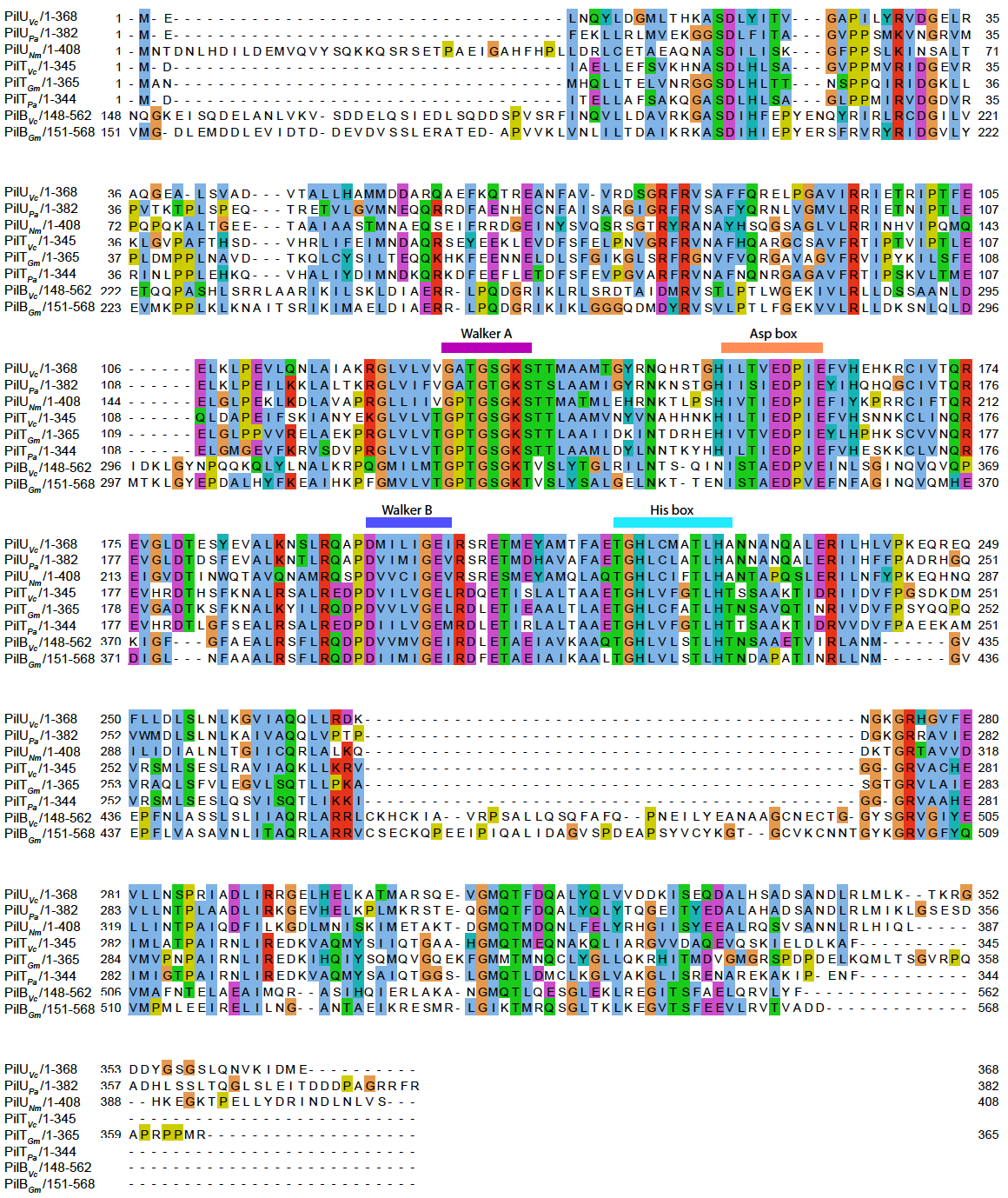
**

**Figure S1. Sequence alignment of PilU, PilT and PilB.**

**
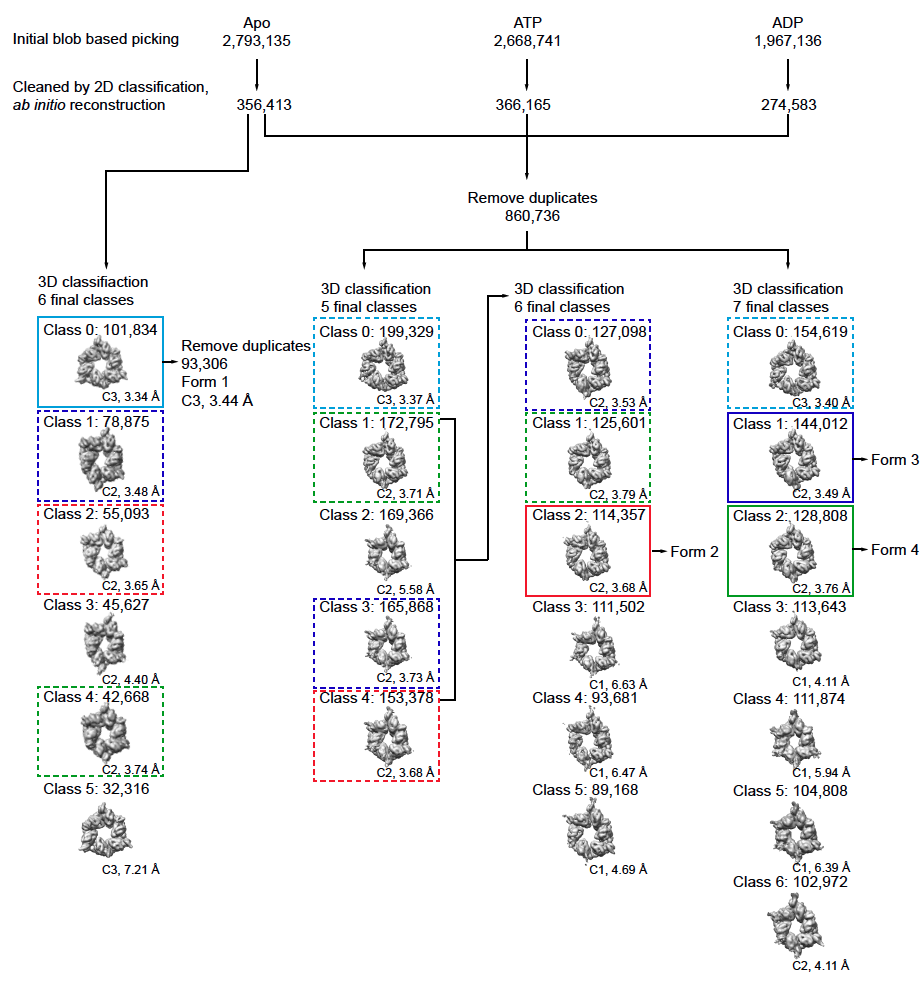
**

**Figure S2. Cryo-EM particle flow.** Three datasets (dataset 1: apo, 3,559 movies; dataset 2: in the presence of ATP, 3,788 movies; dataset 3: in the presence of ADP, 3,836 movies) were collected and processed independently to select clean particles. From dataset 1, 3D classification followed by homogeneous refinement yielded PilU form 1 (C3 symmetry, 3.44 Å, cyan box). Qualitative inspection of 2D class averages showed no obvious nucleotide-dependent differences in overall particle shape, apparent symmetry, or distribution of major views; therefore, particles from all three datasets were pooled for further analysis. Multiple rounds of 3D classification and homogeneous refinement of the combined particle set resolved PilU form 2 (C2 symmetry, 3.68 Å, red box), form 3 (C2 symmetry, 3.49 Å, blue box), and form 4 (C2 symmetry, 3.76 Å, green box). Similar conformations corresponding to PilU forms 1-4 were observed across multiple datasets and 3D classification jobs (cyan dashed box: classes similar to form 1; red dashed box: classes similar to form 2; blue dashed box: classes similar to form 3; green dashed box: classes similar to form 4), further indicate that the addition of ATP and ADP did not substantially change the overall PilU conformations, even if the relative population of particles in each class may differ. For clarity, only the highest-quality reconstruction of each form is used for atomic model building and subsequent analysis. 3D reconstructions with nominal resolutions worse than ~4 Å, which may represent minor conformations or other poorly resolved classes, lacked interpretable structural features and were therefore not assigned as discrete PilU forms and were not further analyzed. Differences in particle numbers across similar reconstructions from individual classification jobs likely reflect the stochastic nature of 3D classification in the presence of continuous heterogeneity and its sensitivity to parameter choices, rather than significant shifts in conformational populations.


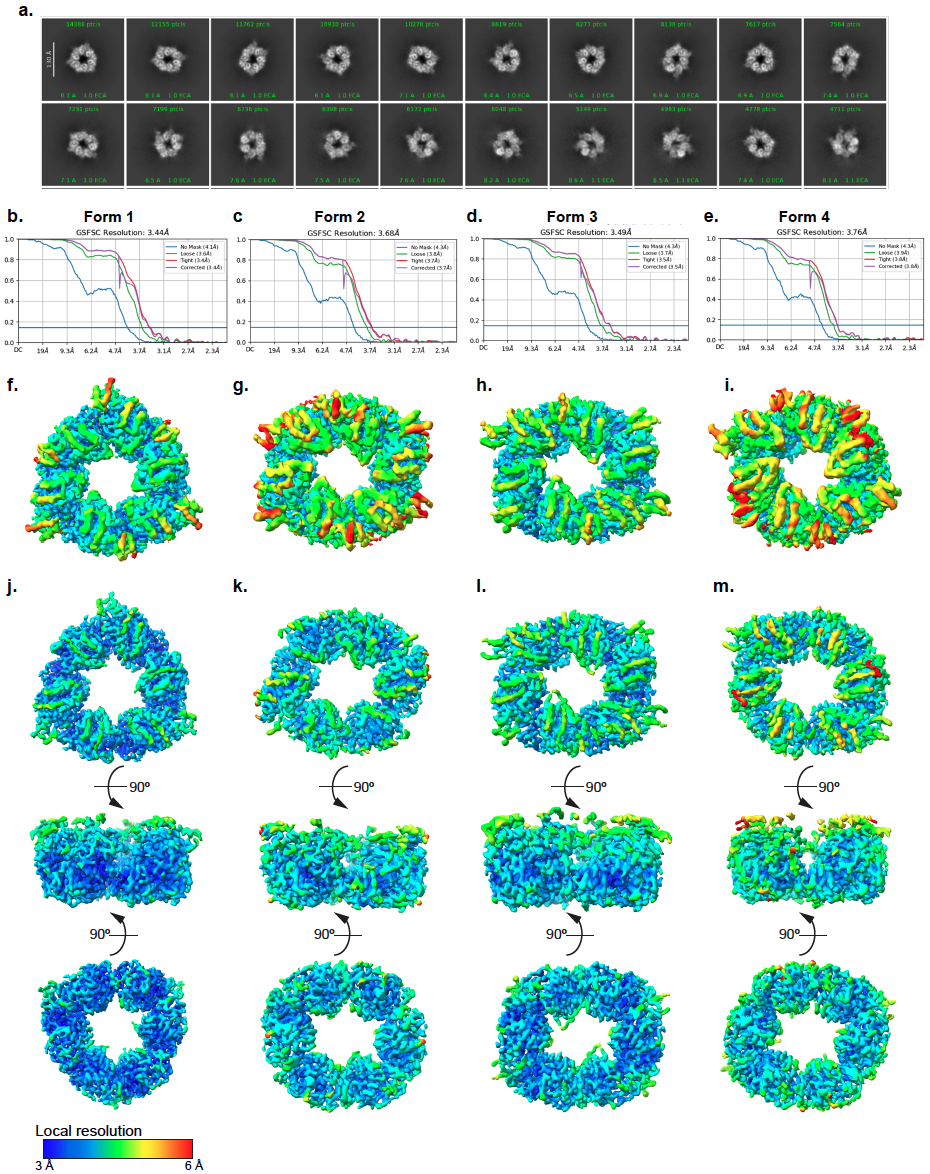


**Figure S3. 2D classes and electron density maps.** a) 2D classes of PilU show different conformations of hexameric rings. b-e) Global FSC of forms 1-4. f-i) Low contour local resolution maps of forms 1-4. j-m) High contour local resolution maps of forms 1-4.


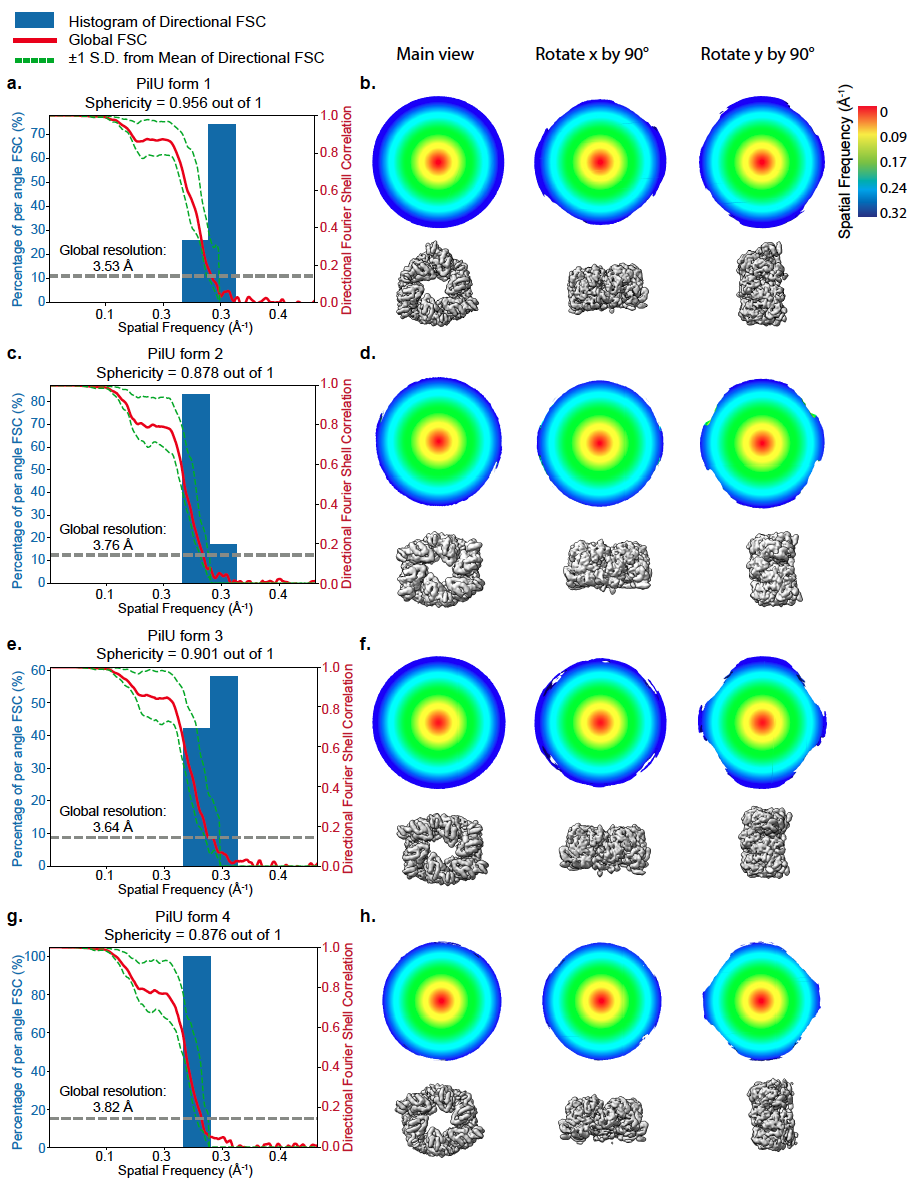


**Figure S4. Resolution assessment of PilU forms 1-4 by directional FSC.** Global and directional Fourier shell correlation curves calculated between half maps with masks used in the final 3D refinement are shown for PilU forms 1-4 (global FSC: solid red line; ±1 standard deviation from mean of directional FSC: dashed green line). Global resolutions estimated by 3DFSC are slightly lower (0.06-0.15 Å) than those estimated by CryoSPARC. Directional resolution anisotropy was assessed by 3D FSC analysis, visualized as FSC volumes rotated by 90° about the x and y axes. The narrow distribution of the per angle FSC histogram and the near-spherical 3D FSC distributions indicate minimal preferred particle orientation and limited resolution anisotropy for all four reconstructions.

**
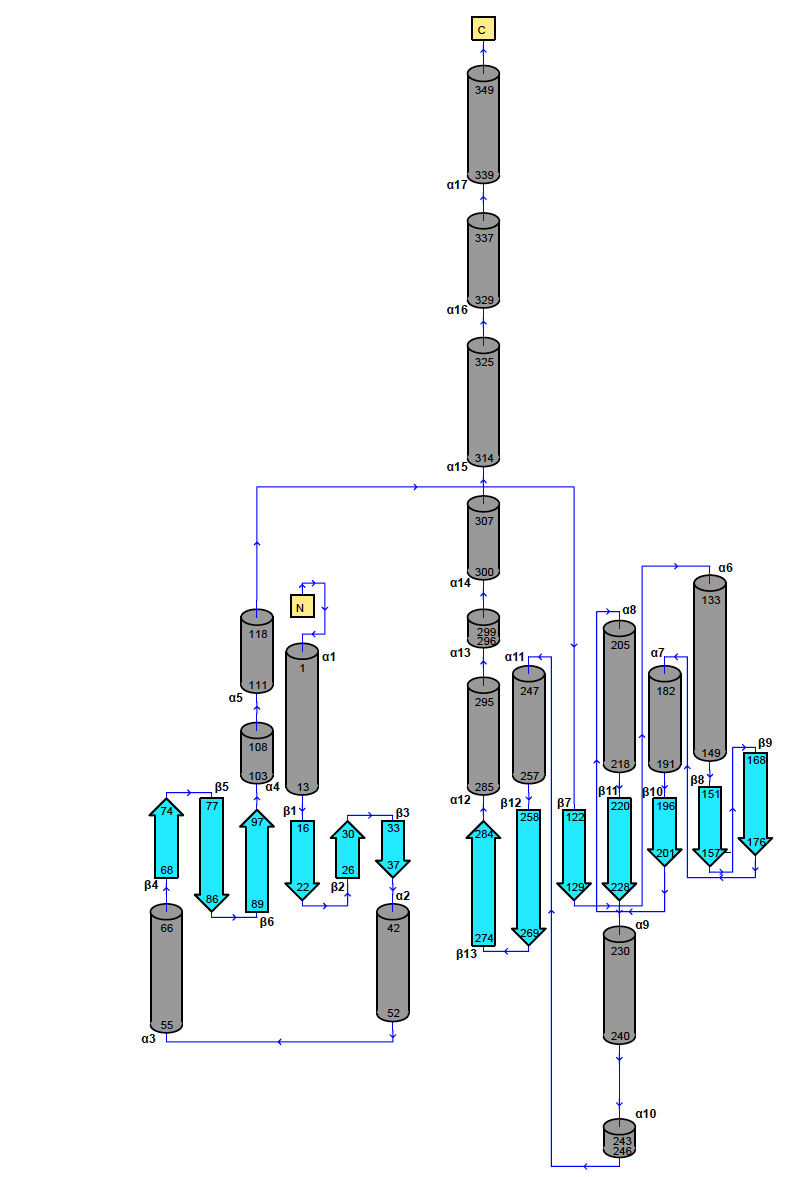
**

**Figure S5. Secondary structure of PilU subunit.**

**
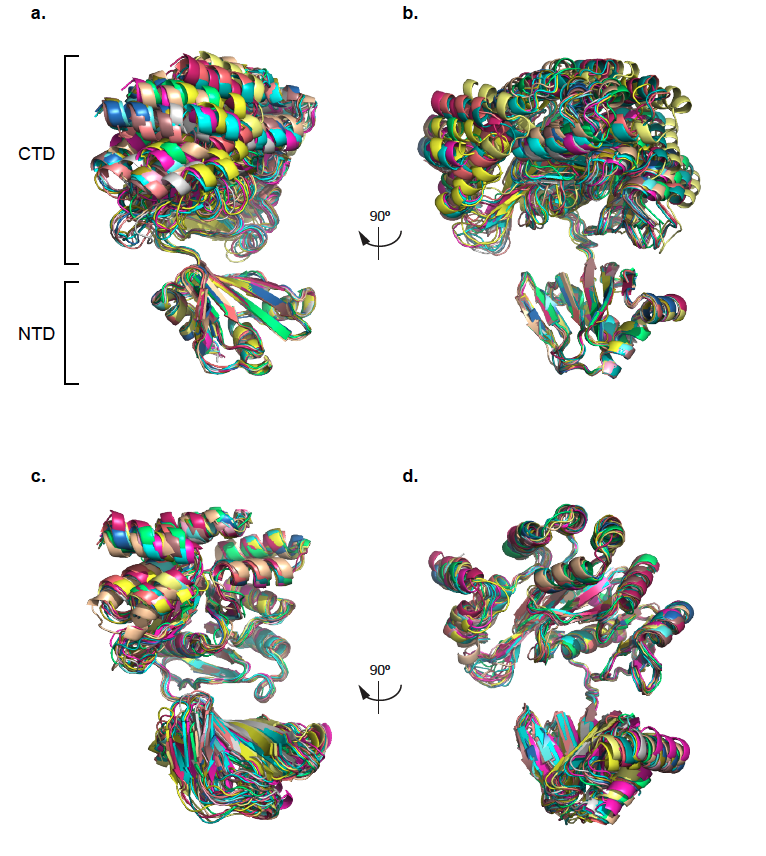
**

**Figure S6. Superimposed PilU subunits.** a, b) PilU subunits superimposed on NTD. c, d) PilU subunits superimposed on CTD.

**
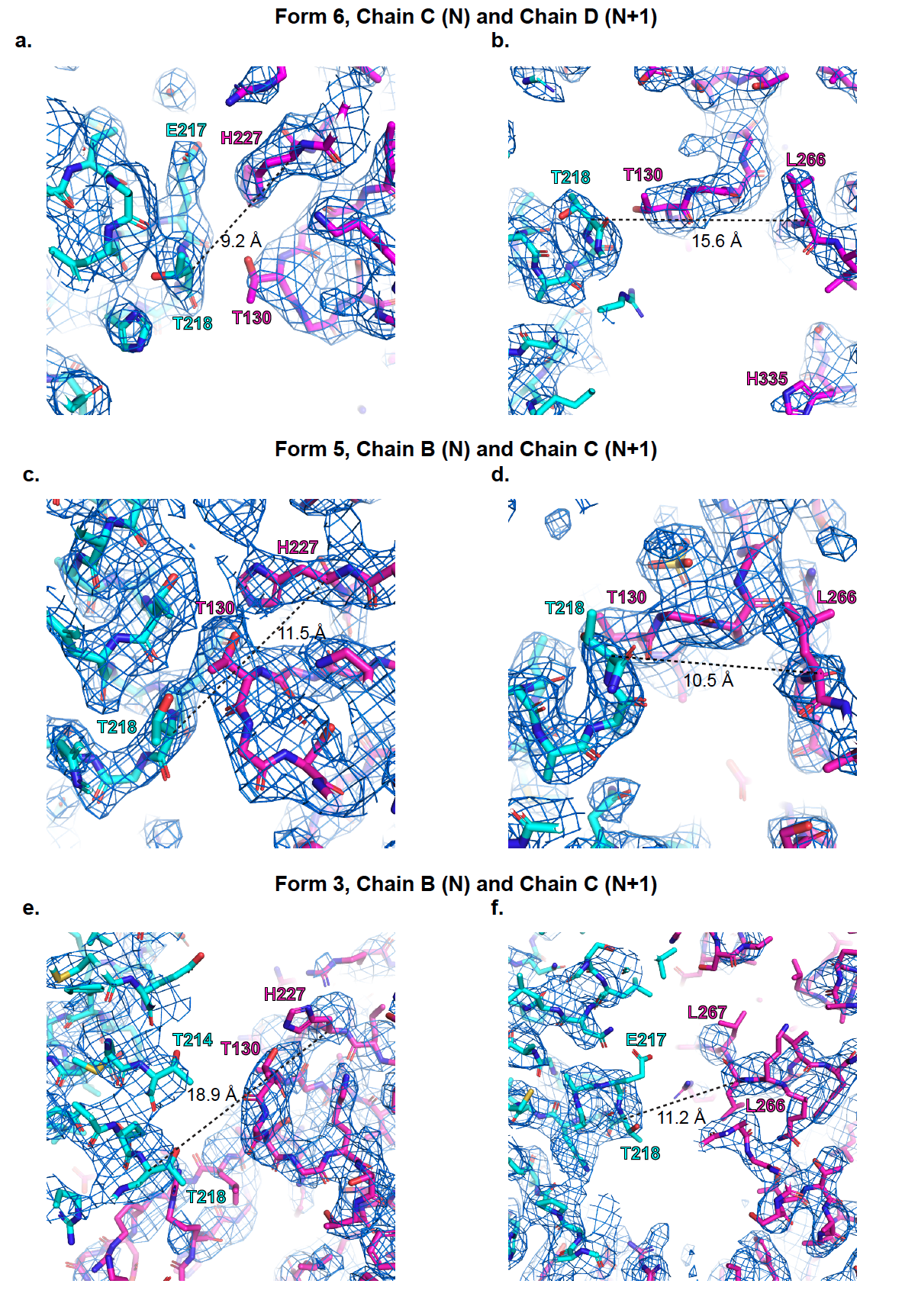
**

**Figure S7. Density map and distance measurement between T218_N_, H227_N+1_ and L266_N+1_ in forms 6, 5 and 3.**

**
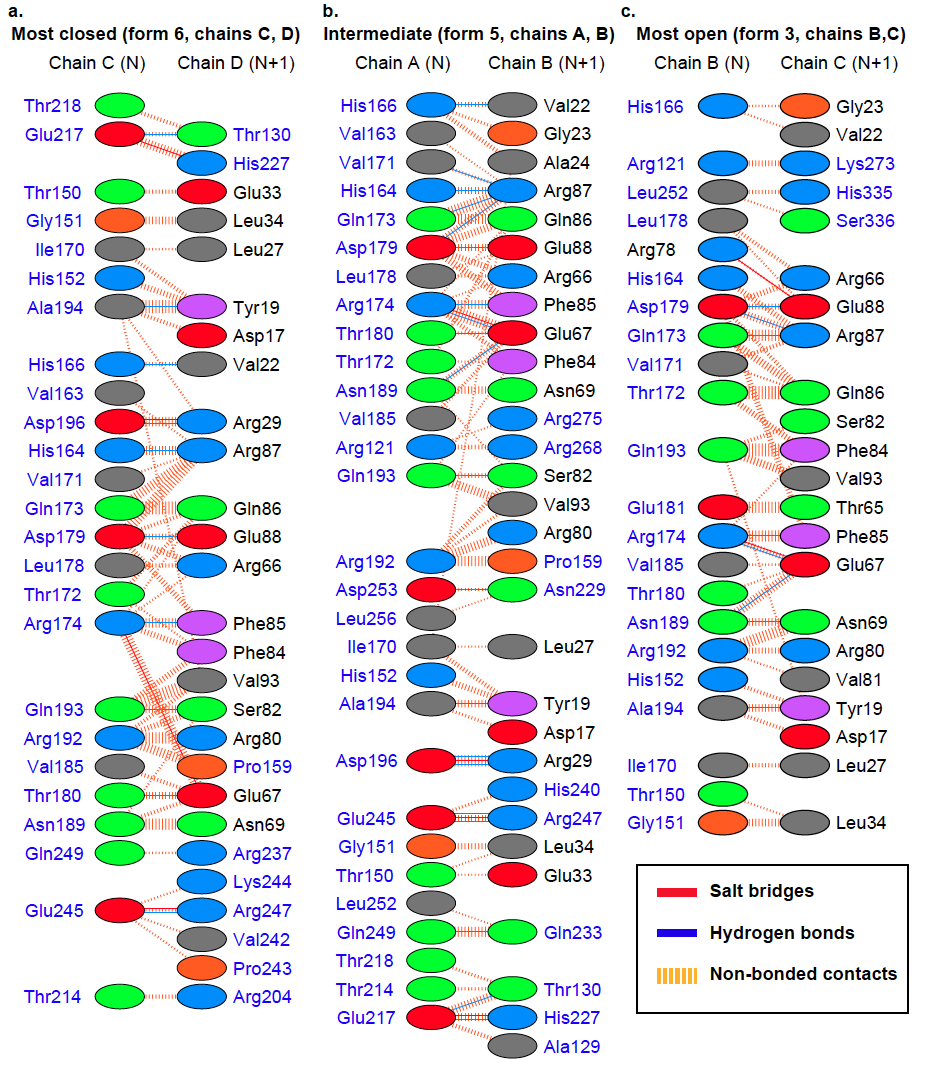
**

**Figure S8. Contacts between PilU subunit interface.** Residues in the NTD are shown in black text, and residues in the CTD are shown in blue text. From the closed to the open state, contacts between CTD_N_ and CTD_N+1_ are significantly reduced.

**
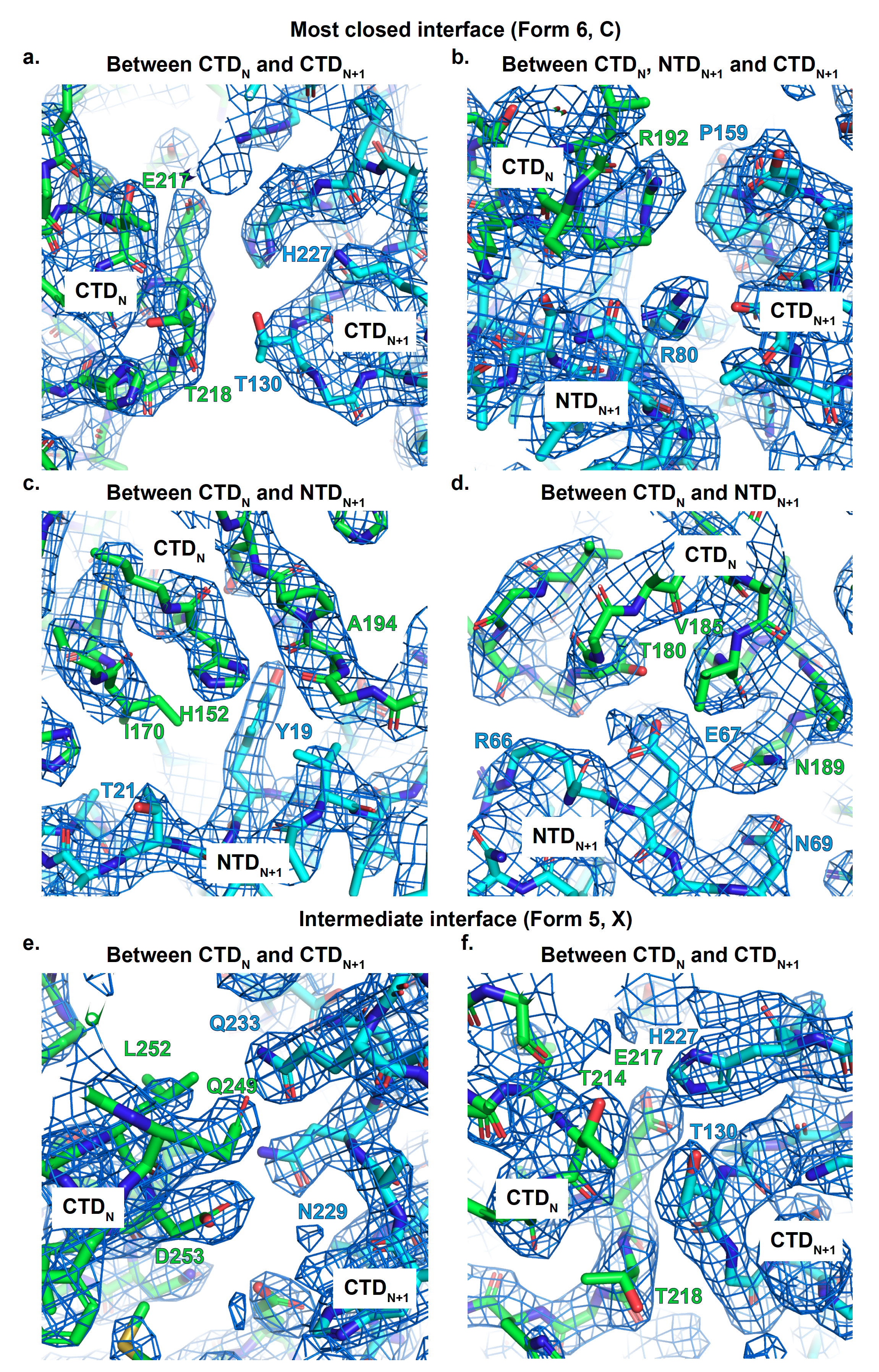
**

**Figure S9. Density at PilU subunit interface.** Chain_N_ is shown in green, and chain_N+1_ is shown in cyan.


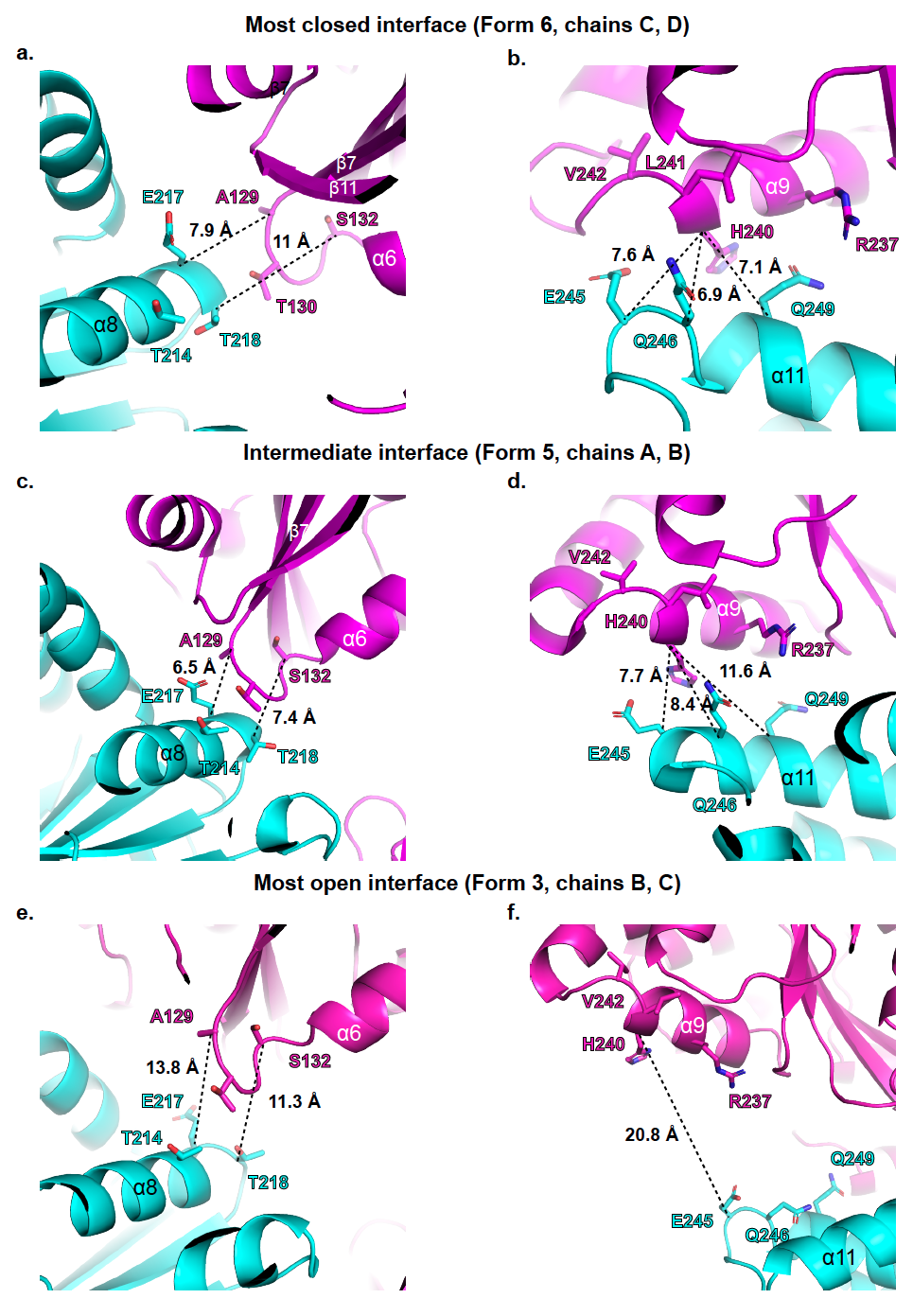


**Figure S10. Distance between key residues at the PilU subunit interface.** Chain_N_ is shown in green, and chain_N+1_ is shown in cyan.
